# Prevalence of *Helicobacter pylori* Infection, Its Virulent Genotypes, and Epstein-Barr Virus in Peruvian Patients With Chronic Gastritis and Gastric Cancer

**DOI:** 10.1200/JGO.19.00122

**Published:** 2019-09-03

**Authors:** Carlos A. Castaneda, Miluska Castillo, Iván Chavez, Fernando Barreda, Nancy Suarez, Jais Nieves, Luis A. Bernabe, Daniel Valdivia, Eloy Ruiz, Emmanuel Dias-Neto, Maria P. Landa-Baella, Yaqueline Bazan, Carlos A. Rengifo, Paola Montenegro

**Affiliations:** ^1^ Instituto Nacional de Enfermedades Neoplasicas, Lima, Peru; ^2^ A.C. Camargo Cancer Center, São Paulo, Brazil

## Abstract

**PURPOSE:**

*Helicobacter pylori* (HP) and Epstein Barr virus (EBV) infections induce chronic gastritis (CG) and are accepted carcinogenics of gastric cancer (GC). Our objective for this study was to determine the prevalence of these agents and clinicopathological features of GC and CG associated with the infection.

**PATIENTS AND METHODS:**

A single-center cohort of 375 Peruvian patients with GC and 165 control subjects with CG were analyzed. Evaluation of HP and EBV genes was performed through quantitative polymerase chain reaction.

**RESULTS:**

Prevalence of HP was 62.9% in the whole population and 60.8% in the GC subset. The *cagA* gene was detected in 79.9%; *vacAs1* and *vacAm1* alleles in 41.6% and 60.7%, respectively; and concurrent expression of *vacAs1* and *vacAm1* in 30.4% of infected patients in the whole series. The prevalence of EBV was 14.1% in the whole population and was higher in GC (*P* < .001). Coinfection of HP and EBV was found in 7.8% and was also higher in GC in univariate (*P* < .001) and multivariate (*P* = .011) analyses. Infection rates of HP and EBV were not associated with a geographic location in the whole series. Few clinicopathological features have been associated with infectious status.

**CONCLUSION:**

Prevalence of HP infection and virulent strains are high in the Peruvian population. Infection by EBV was more frequent in patients with GC.

## INTRODUCTION

There are large regional differences in gastric cancer (GC) rates, and the highest incidence rates are in East Asia and South America. More than two-thirds of cases occurred in middle- and low-income countries, according to GLOBOCAN 2018.^1^ In addition, areas with the highest GC rates have been described within countries.^2,3^

In the Andean country of Peru, GC is the third most common cancer and causes the highest absolute number of cancer deaths.^4^ Persistent infection with *Helicobacter pylori* (HP) induces chronic inflammation and gastric carcinogenesis. HP infection in gastric mucosa has been associated with low socioeconomic status, and its prevalence in adults living in developing Latin American countries is 70% to 80%.^5-7^

The genomes of HP are heterogeneous and encode different virulence factors that play an important role in the clinical outcome of the infection. Cytotoxin-associated gene A (cagA) is a protein encoded by the *cagA* gene. It translocates to epithelial cells and activates signaling pathways that induce cellular changes and the production of proinflammatory cytokines. The vacuolating cytotoxin A (vacA) of HP is encoded by the *vacA* gene, causes direct damage to the gastric epithelium, and stimulates an acute inflammatory process. The *vacA* gene has the s1 or s2 allele types in the signal region (s) whereas the m1 and m2 allele types are in the middle region (m). The combination of s1/m1 allele types results in high levels of cytotoxin. The proinflammatory potential of *cagA*-positive and *vacA* allele variants of HP may explain HP’s association with severe atrophic gastritis, peptic ulcer, and gastric adenocarcinoma.^8^

Gastric infection by Epstein-Barr virus (EBV) produces a severe mucosal inflammatory response, has demonstrated carcinogenic activity, and leads to a specific subtype of GC.^9,10^ Recent studies suggest that HP and EBV coinfection could have a synergistic carcinogenic effect and would be more frequently found in GC than chronic gastritis (CG).^11-13^

**CONTEXT**

**Key Objective**
The purpose of this study was to analyze the prevalence of HP and EBV infections in Peruvian patients with chronic gastritis and gastric cancer, and to evaluate clinicopathological features associated with the presence of the infectious agents.
**Knowledge Generated**
The infection rate of H. pylori is higher than 60% in our population, and most strains were cagA positive. Epstein-Barr virus infection was more frequent in gastric cancer than chronic gastritis, and its coinfection with H. pylori was also more frequently found in gastric cancer.
**Relevance**
Treating H. pylori infection in South American countries should be enforced in public health care to reduce gastric cancer rates. To our knowledge, the current study is one of the largest series of molecular epidemiology of infectious carcinogenic agents in gastric samples from one South American country. We expect our results can influence public policies in South American countries.

The prevalence of the genotypes of HP that express the most virulent factors and EBV change with geographic area, and there is limited information about rates of these infections in the Peruvian population. Information about prevalence differences of these infectious agents is even lower among the three Peruvian recognized regions: coast, highland and, rain forest. The coast (11.7% of the national territory) is the most urbanized area, has the highest population (56.3% of the total population), and is where the largest city in Peru, the crowded and capital city of Lima, is located. The Peruvian rain forest (Peruvian Amazon) represents 60.3% of the Peruvian territory but only 14.0% of the total population^15^.

The purpose of this study was to analyze the prevalence of HP and EBV infections in Peruvian patients with CG and GC, and to evaluate clinicopathological features associated with the presence of the infectious agents.

## PATIENTS AND METHODS

**FIG 1:**
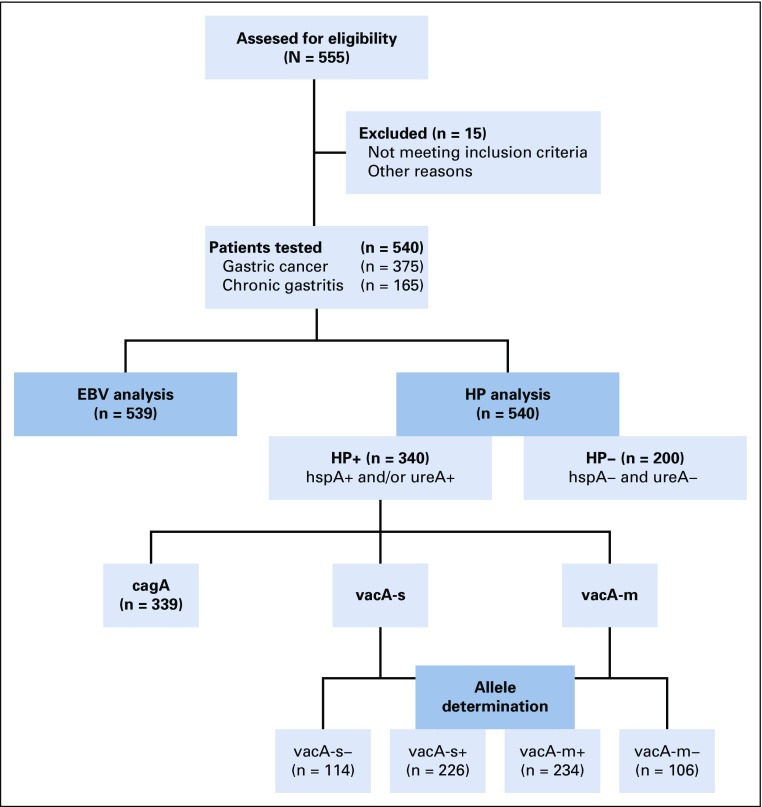
Flowchart showing screened patients with gastric cancer and chronic gastritis, and subgroups with allele determination. EBV, Epstein-Barr virus; HP, *Helicobacter pylori*.

We developed a cross-sectional study with 540 patients who came to the Instituto Nacional de Enfermedades Neoplasicas (INEN), Lima, Peru, for screening or GC treatment from 2015 to 2018. We included 375 patients with GC who underwent diagnostic endoscopy (n = 67) or gastrectomy (n = 308) and 165 patients with CG who underwent screening endoscopies (nonmalignancies). Inclusion criteria were histology of adenocarcinoma and not being previously exposed to chemotherapy, in the cancer group. CONSORT methodology was followed in the study development (Fig 1).

The Human Subjects Committee of INEN approved the conduct of this study (Protocol No. 050-2015-CIE/INEN). Before their inclusion, patients received information regarding the purpose and the conduct of the study. Patients gave written consent for the storage of their samples at the INEN biobank and for their samples’ use in research.

We collected clinicopathological data from the INEN’s patient files and from an epidemiologic questionnaire, including the patient’s home address. Peru is divided into 25 provinces distributed among the three previously mentioned regions. In addition, Lima city is divided into districts and they were settled into North (seven districts), South,^7^ Center,^15^ and East^7^ sectors. Callao (the main harbor of the country) shares limits with Lima city and was considered for the Lima sectors analysis.^15,16^

### Endoscopy and Histology

Biopsy specimens were obtained from tumoral, proximal, and distant nontumoral tissues in patients with GC and from only one area from those with CG. Samples were immediately saved and frozen at −70°C until processing.

### Detection of HP Infection

Diagnosis of HP was accepted when at least the results of one of the areas were positive. The detection of HP in gastric tissue samples was carried out through the extraction of genomic DNA with the GeneJETGenomic DNA kit (Thermo Fisher Scientific, Waltham, MA), the quantification by fluorometry was conducted using the Quantus fluorometer (Promega, Madison, WI), and, subsequently, the standard detection of the colonizing genes (*hspA* and *UreA*) by quantitative polymerase chain reaction (qPCR) in the LightCycler 96 Instrument Thermal Cycler (Roche, Mannheim, Germany). The hspA gene sequence was detected with the following primers: reverse: 5′-GCT ATC TGA AAA TTT GAT TTC TTT TGC-3′ and forward: 5′-TGC GCT ATA GTT GTG TCG C-3′; and that of *UreA*: reverse: 5′-TTG TCT GCT TGT CTA TCA ACC-3′ and forward: 5′-GAG AAT GAG ATG AAA CTC ACC C-3′. The mixture for the qPCR was composed of PCR-grade water, Sybr GreenFastStartEssential DNA Green Master (Roche), the respective primers, and the sample of genomic DNA.^17,18^

### Evaluation of HP Virulence Genes

Virulence of *cagA*, *vacAs*, and *vacAm* genes was tested in HP-positive patients detected by constitutive genes (*hspA* and *ureA*). The DNA of HP was detected with qPCR using oligonucleotides directed to the *hspA* and *UreA* genes. The signal and middle regions of *vacA* were genotyped by qPCR using the oligonucleotides previously described by Camorlinga-Ponce et al.^18a^

The *cagA* gene sequence was detected with the following primers: reverse: 5′-TCT​AAT​CCT​GTT​TGC​TCC​CCA-3′ and forward: 5′-CTC​ATT​GCG​AAG​GCG​ACC​T-3′. The *vacAs* gene was detected with the following primers: reverse: 5′-GCG​TCA​AAA​TAA​TTC​CAA​GG-3′ and forward: 5′-CAA​TCT​GTC​CAA​TCA​AGC​GAG-3. And to detect the *vacAm* gene, the following primers were used: reverse: 5′-CTG​CTT​GAA​TGC​GCC​AAA​C-3′ and forward: 5′-ATG​GAA​ATA​CAA​CAA​ACA​CAC-3′.^17,18^

The amplification program included one cycle at 95°C for 10 minutes; 45 cycles at 95°C for 15 seconds, 57°C to 63°C (depending on the gene) for 15 seconds, and 72°C for 20 seconds; and a melting curve 95°C for 10 seconds, 65°C for 60 seconds, and 97°C for 1 second.^19,20^

A melting curve for *vacA* alleles was constructed for each primer pair to verify the presence of one gene-specific peak and the absence of primer dimmer. Genotype *s1/m1* was identified in positive patients for gene expression by comparing them with the gene-specific peak obtained from strain ATCC700392D-5 HP *vacA s1+m1+*.

### Evaluation of the EBV Gene

EBV qPCR was performed on DNA using the Primerdesign EBV (human herpesvirus 4) kit (genesig Advanced, Southampton, UK) with a region of *BNRF1* as the target. ;The target sequence within the *BNRF1* gene was a good genetic marker for EBV in other clinical real-time–PCR-based studies.^21^ The amplification program included one cycle at 95°C for 120 seconds, 50 cycles at 95°C for 10 seconds, and 60°C for 10 seconds.

### Statistical Analysis

We used Pearson χ^2^ or Fisher’s exact tests to compare frequencies between groups. Kruskal-Wallis test was used for comparing number of copies HspA and UreA among tumoral, and proximal and distal nontumoral areas. Multivariate analysis was performed using a multiple logistic regression model for factors associated with GC compared with the CG subset and results were evaluated with odds ratios. *P* < .05 was considered statistically significant. We used the R, version 3.4.1 (https://www.r-project.org/), for the statistical analyses.

## RESULTS

### Patients and Histologic Diagnosis

In the whole series, the median age was 60 years and 57.4% of the patients were women. Most came from the coast region (69.4%), and most people from Lima city came from central districts (28.6%).

In the group of patients with GC, most patients were of poorly and undifferentiated grade (57.8%), and had intestinal histology (47.6%), lymphovascular invasion (69.1%), and perineural invasion (46.1%). The most common clinical stages were III and IV (65.7%). Most patients had antrum involvement (62.6%) and evaluated samples came from biopsy (17.9%), or subtotal (51.5%) or total gastrectomy (30.7%). In the CG group, most patients were women (78.8%) and were at least 60 years old (33.2%). Metaplasia was found in 26.7% (Table 1).

**TABLE 1:**
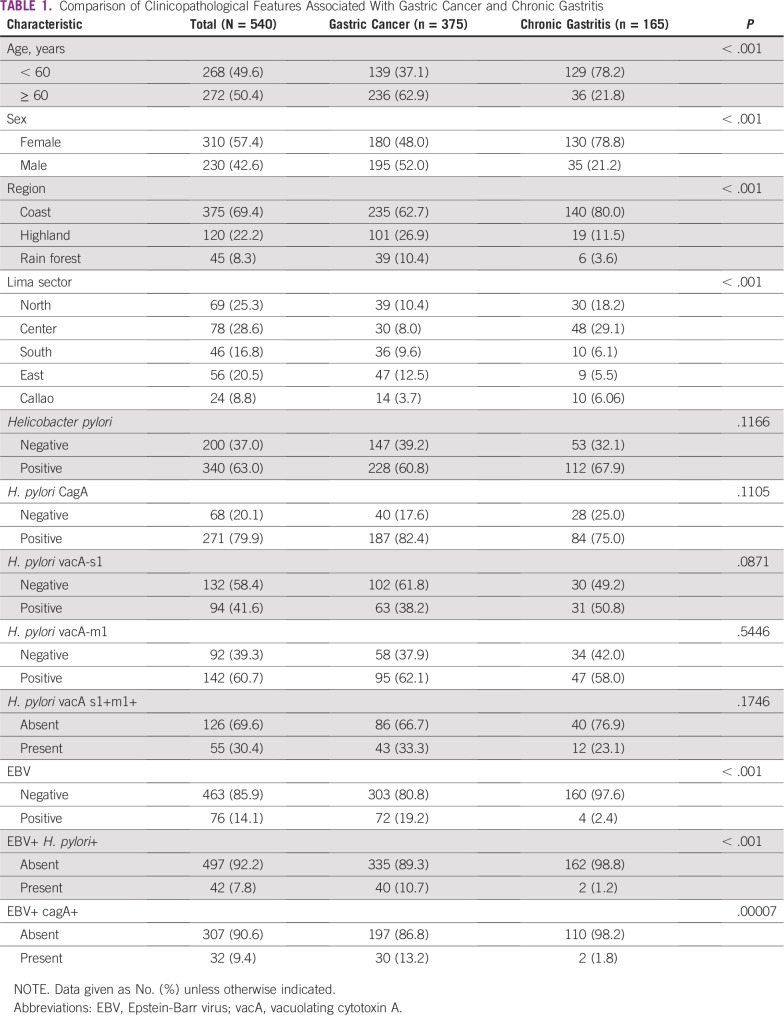
Comparison of Clinicopathological Features Associated With Gastric Cancer and Chronic Gastritis

### Prevalence of HP

The prevalence of HP was 62.9% in the whole population and 60.8% in GC subset. In the GC subset, the infection was detected in tumoral, proximal, or distal areas in 46.0%, 53.2%, and 49.5% of patients, respectively. The highest median number of *ureA* copies/μL was found in nontumoral regions (95.5 in tumoral, 149.6 in proximal, and 142.8 copies/μL in distal areas; *P* < .001). The highest median number of *hspA* copies/μL was found in nontumoral regions (220.9 in tumoral, 608.5 in proximal, and 632.2 copies/μL in distal areas; *P* = .003).

Infection prevalence in the coast (n = 239 of 375; 63.7%), highland (n = 70 of 120; 58.3%), and rain forest (n = 31 of 45; 68.8%) areas were similar in the whole series (*P* = .39). There were no associations between the presence of HP and age (*P* = .45), sex (*P* = .12), cancer stage (*P* = .059), or other evaluated clinicopathological features (*P* > .05) in the GC group.

### Prevalence of HP With *cagA* Genotype

The *cagA* gene was evaluable in 339 samples and detected in 79.9% of HP-infected patients in the whole series (Fig 1). The protein, cagA, tended to be more frequent in GC than CG (82.4% *v* 75%; *P* = .12; Table 1). In the GC subset, presence of *cagA* was not associated with age (*P* = .12), sex (*P* = .49), cancer stage (*P* = .69), or other evaluated clinicopathological feature (*P* > .05; Table 2).

**TABLE 2:**
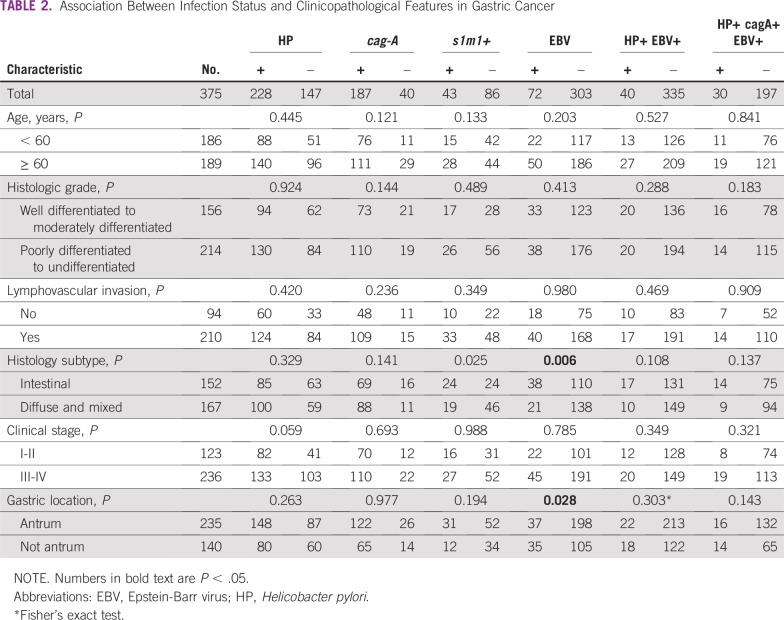
Association Between Infection Status and Clinicopathological Features in Gastric Cancer

### Prevalence of HP With *vacA* Alleles

*VacAs* was detected in 226 and *vacAm* in 234 patients (Fig 1). *VacAs1* and *vacAm1* alleles were detected in 41.6% and 60.7%, respectively, in the whole series, and in 38.2% and 62.1%, respectively, in the GC subset. There was a correlation between presence of *vacAs1* and both *vacAm1* (*P* < .001) and *cagA* (*P* < .001). Concurrent expression of *vacAs1+m1+* was found in 30% in the whole series and 33.3% in the GC subset (Table 1). In the GC subset, *s1+m1+* was associated with intestinal histology (*P* = .03; Table 2).

### Prevalence of EBV Infection

The prevalence of EBV was 14.1% in the whole population and was higher in the GC than in the CG subset (19.2% *v* 2.4%; *P* < .001; Table 1). Infection with EBV was associated with intestinal histology (*P* = .006) and nonantrum location (*P* = .028) in the GC subset (Table 2). Coinfection of HP and EBV was found in 42 patients (7.8%) in the whole series and was higher in patients with GC than in the subset with CG (10.7% *v* 1.2%; *P* < .001). Coinfection of HP *cagA+* and EBV was found in 9.4% of patients and was higher in the GC group than in the CG subset (13.2% *v* 1.8%; *P* < .001). There was no association between presence of any infection and metaplasia in the CG subset (*P* > .05; Table 3). According to multivariate analysis, EBV+ HP+ was present seven times more frequently in patients with GC than was EBV− HP− (*P* = .011; Table 4).

**TABLE 3:**
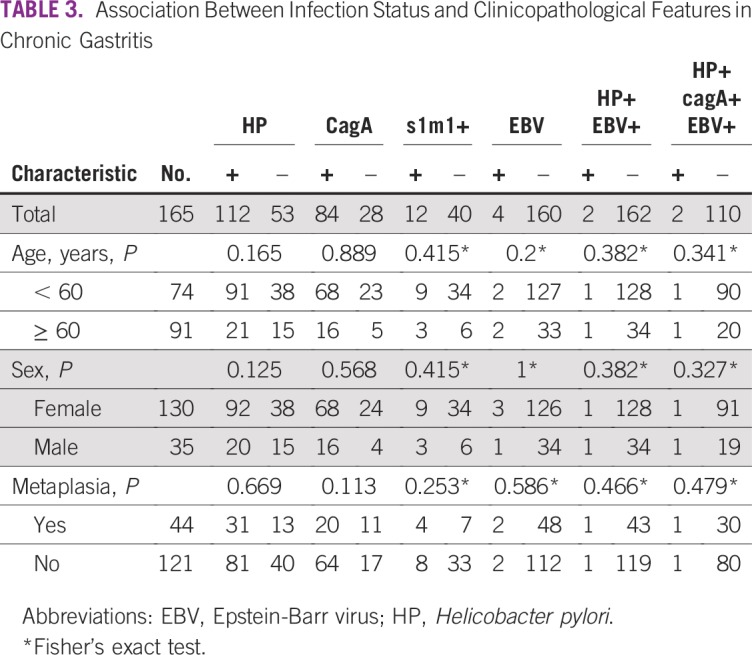
Association Between Infection Status and Clinicopathological Features in Chronic Gastritis

**TABLE 4:**
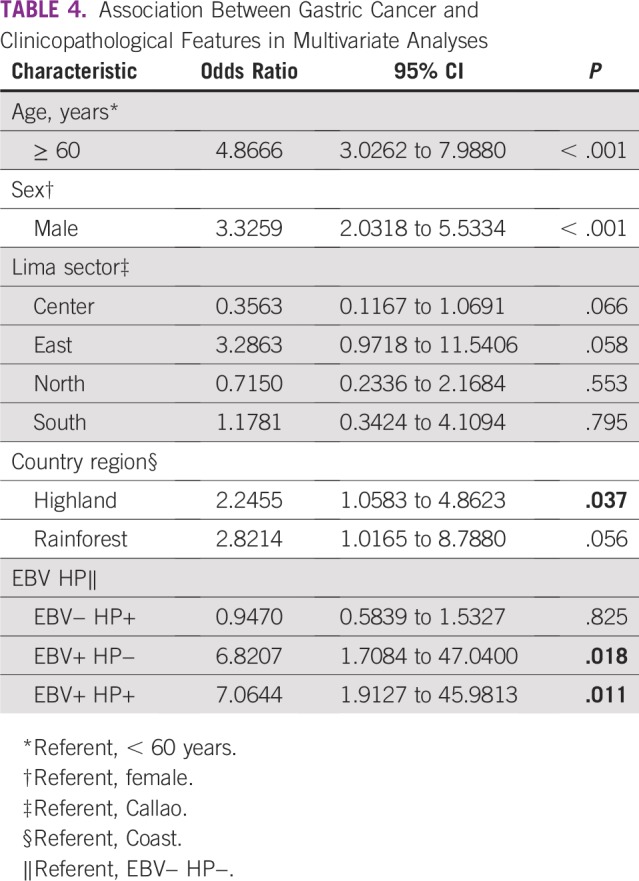
Association Between Gastric Cancer and Clinicopathological Features in Multivariate Analyses

## DISCUSSION

The rates of HP infection in patients with GC (60.8%) in this study is similar to the reported prevalence in developing countries and similar to CG samples (67.9%). This prevalence is much lower than the prevalence reported three decades ago. This phenomenon of reduction has also been described in developed countries^22-24^ and could be related to low rates of HP contamination in drinking water because of an improvement in water potability, as recently described by Boehnke et al,^25^ as well as the increased use of antibiotics.^24,26,27^

Studies have described HP infection rates that differed among patients living in the coast, highland, and rain forest areas; this has been suggested to be as a result of higher HP infection rates in people living in high-altitude areas compared with those at sea level.^22,24,28,29^ Influence of racial factors in this phenomenon is probably not important, because a previous study did not find differences in HP infection rates among the Peruvian population and the Japanese colony resident in Peru (The Gastrointestinal Physiology Working Group of the Cayetano Heredia and the Johns Hopkins University, 1992).^23^ We found no difference among those patients from any of these areas in our whole series.

When we analyzed only patients from Lima city, we found that the districts with higher HP infection rates are different to those located in the areas previously described as GC rates.^4^ These differences, in addition to the absence of similarity between Peruvian regions with higher rates of HP infection and those with higher rates of GC diagnosis in our series and previous studies,^22-24,28,29^ could be related to the fact that patients from highland and rain forest regions constantly visit coastal cities, because the latter have better services, such as the Health Care Institute where diagnostics are conducted, and by a similar HP contamination rates of drinking water we have found in different districts (unpublished data). However, we cannot exclude that the differences could be related to the small sample size in our study.

We found that 79.9% of the HP strains in our whole series were *cagA+.* A previous study reported that more than 90% of HP strains in Lima were *cagA+*.^22^ The frequency we report is lower than that reported in a northeastern region of Brazil (96.7%)^30,31^ but higher than that reported in Mexico (47.6% to 73.9%).^12,32-34^ Differences in regional distribution of strains are clear: The frequency of *cagA*-positive HP is 90% to 95% in Asian countries, whereas only it is 50% to 60% in Western countries^5,34a,35^; however, we did not find differences in cagA+ infection rates among Peruvian regions nor among Lima sectors. Although *cagA*+ strains have been associated with strong carcinogenic activity,^34b^ we did not find higher rates in GC samples compared with CG samples, which indicates that presence of aggressive strains of HP is not enough to develop GC. Our results can be explained by the fact that cancer is a long-term result of HP infection and it starts to disappear as hypochlorhydria sets in.^35-37^Prevalence of HP *vacA s1+m1+* strains was 33.3%.This rate is similar to that reported in southern Mexico (38.5%)^38^ but lower than frequencies found in other regions of Mexico, Colombia, and Brazil.^31,32,34^ This strain has been described as more aggressive; however, we did not find higher rates in GC samples compared with CG samples.

On the other hand, 14% of gastric tissues had EBV infection in our whole series and the prevalence was 19.2% in the GC subset. These rates are higher than the previously reported rate of 3.9% (diagnosed by in situ hybridization) in a Lima population and close to the average rate of 10% EBV infection in patients with GC in South American countries.^13,39-41^ We selected qPCR instead of in situ hybridization because the former can detect fewer gene copies.^42^ A coinfection of EBV and HP was found in 7.8% of patients in the whole series and was also more frequent in the GC group than in the CG subset (*P* < .001). Furthermore, multivariate analysis showed the concurrence was seven times more frequently associated with GC than in patients negative for both EBV and HP infection. Rates of coinfection in our study were lower than those reported in two Mexican series; however, both series found that coinfection of HP and EBV was more frequent in patients with GC than in those with CG.^11-13^

The association between coinfection of HP and EBV with gastric malignancy could be explained by the preclinical finding of a synergy of both pathogens to induce carcinogenesis. Double infections of HP and EBV can synergistically act to enhance expression of IL-17 and maintain an inflammatory state that more severely damages gastric mucosa. HP also induces monochloramine generation, which could reactivate EBV.^11,12,43-46^ To our knowledge, the current study is one of the largest series of molecular epidemiology of infectious carcinogenic agents in gastric samples from one South American country. To produce confident values, the presence of HP infection was evaluated in tumoral and nontumoral regions; correlation of detection was high, but more copies of constitutive genes were found in distal nontumoral regions, probably as a result of environmental challenges generated by malignant cells over bacteria growth, like pH alteration. We expect our results can influence public policies in South American countries.

A weakness of our study is that we included every patient with CG or GC who came to the Institute for diagnosis in a convenience sample instead of paired selection. However, the multivariate analysis allowed us to better quantify the association with cancer compared with nonmalignant pathology for every feature.

In conclusion, our results indicate that HP infection and its virulent strains are frequent and widely spread among Peruvian regions. EBV and dual infection by HP and EBV are more frequent in patients with GC than in those with CG.
